# Optical Fibre-Based Sensors for Oil and Gas Applications

**DOI:** 10.3390/s21186047

**Published:** 2021-09-09

**Authors:** Jincy Johny, Solomon Amos, Radhakrishna Prabhu

**Affiliations:** 1 School of Engineering, Robert Gordon University, Aberdeen AB10 7GJ, UK; jincy.tist@gmail.com; 2 Department of Computer Science, Deramore Lane, University of York, Heslington, York YO10 5GH, UK; solomon.amos@york.ac.uk

**Keywords:** optical fibre, oil and gas, sensor

## Abstract

Oil and gas (O&G) explorations moving into deeper zones for enhanced oil and gas recovery are causing serious safety concerns across the world. The sensing of critical multiple parameters like high pressure, high temperature (HPHT), chemicals, etc., are required at longer distances in real-time. Traditional electrical sensors operate less effectively under these extreme environmental conditions and are susceptible to electromagnetic interference (EMI). Hence, there is a growing demand for improved sensors with enhanced measurement capabilities and also sensors that generates reliable data for enhanced oil and gas production. In addition to enhanced oil and gas recovery, the sensing technology should also be capable of monitoring the well bore integrity and safety. The sensing requirements of the O&G industry for improved sensing in deeper zones include increased transmission length, improved spatial coverage and integration of multiple sensors with multimodal sensing capability. This imposes problems like signal attenuation, crosstalks and cross sensitivities. Optical fibre-based sensors are expected to provide superior sensing capabilities compared to electrical sensors. This review paper covers a detailed review of different fibre-optic sensing technologies to identify a feasible sensing solution for the O&G industry.

## 1. Introduction

In recent years, the recovery of hydrocarbons has become more and more difficult and challenging as exploration and production operations tend to seek new fronts into deep and ultra-deep harsh environments. As the energy demand continues to rise, there is a need for efficient management and optimization for production operations and systems to make this growing energy demand sustainable. This requires real-time monitoring of long and deep oil wells.

Sensors and sensing data are vital elements in the oil and gas (O&G) industry. O&G exploration and production have been moving into unconventional depths (more than 3 km) in order to meet the growing demand for energy [1]. This results in harsh and extreme operating conditions, which are reflected by critical parameters like temperature, pressure, strain, etc. Therefore, reliable sensors which are able to continuously monitor current down-hole conditions have become very important in managing O&G reservoirs and wells [2]. For efficient O&G resource management and enhanced oil and gas recovery, real-time and dynamic monitoring technologies are required [3,4]. In order to satisfy this need, multi-point or distributed and multimodal simultaneous measurements will be advantageous for drilling and O&G production monitoring.

Exploration and production process monitoring helps to prevent or detect health and safety issues and to significantly enhance O&G production [5]. Detecting and forecasting the conditions of the well at earlier stages have a considerable impact on Health, Safety and Environment (HSE), risk management, well control and cost-control strategies [6]. It enables the oil well technicians and managers to take correct decisions in a timely manner [7]. Continuous sensing and monitoring of unstable parameters like high temperature, pressure, strain, etc., are required in the O&G sector in order to protect and safeguard their valuable assets operating in the harshest and most challenging environments.

Current O&G sensing techniques are mainly based on electrical sensors, which have many constraints when used in adverse environmental conditions [8]. These electrical sensors offer limited performance down-hole and are less reliable for real-time remote monitoring and control. Unfortunately, O&G reservoirs exhibit some of the harshest and least accessible environments on Earth [9,10]. Increasing exploration depth results in High-Pressure High Temperature (HPHT) field conditions, which corresponds to temperatures above 205 °C/400 °F and pressures more than 138 MPa/20,000 psi [11,12]. In such hostile habitats, conventional sensors either experience failure or operate poorly. This occurs mainly due to their inability to withstand high temperature and pressure, as well as corrosive and erosive environmental conditions, found within the oil wells. Other limitations of traditional electrical gauges include limited sensing range, single-point sensing capability and their inability to give continuous monitoring which makes them unsuitable for oil well real-time monitoring applications. In addition, their poor Signal to Noise Ratio (SNR) due to electromagnetic interference (EMI) and their considerable size makes them highly undesirable for in-well applications [13]. On this account, improved technologies are to be developed to retrieve well information, in order to safely maximize oil productivity and reduce exploration and production cost, especially in the present situation of reduced crude oil prices.

Fibre-optic sensing technology can overcome the aforementioned limitations of their electrical counterparts, mainly due to their small size, electrical isolation, corrosion resistance, immunity to EMI and capability to operate in extreme environmental conditions [14,15]. The small size of fibre-optic sensors facilitates them to be safely employed over longer distances with little or no future maintenance [16]. Moreover, they have a reduced risk of failure when exposed to water or other reservoir and pipeline fluids and, also, they do not have any electrical power requirements at the sensor head [17,18]. Another important advantage of fibre sensors is that the same optical fibre can handle dual functions. It can act as the sensing element for physical parameter measurement as well as the transmission medium for the sensed signal. This feature helps in the monitoring and sensing of different O&G critical parameters from remote locations [19]. Furthermore, by exploiting the wavelength multiplexing capability of optical fibre, multi-point or distributed and multi-modal simultaneous measurements can be easily accomplished on the same fibre [20]. Considering all these advantages, optical fibre sensing technology offers an attractive alternative to traditional electrical sensing technology for permanent monitoring of oil well reservoirs.

**Figure 1:**
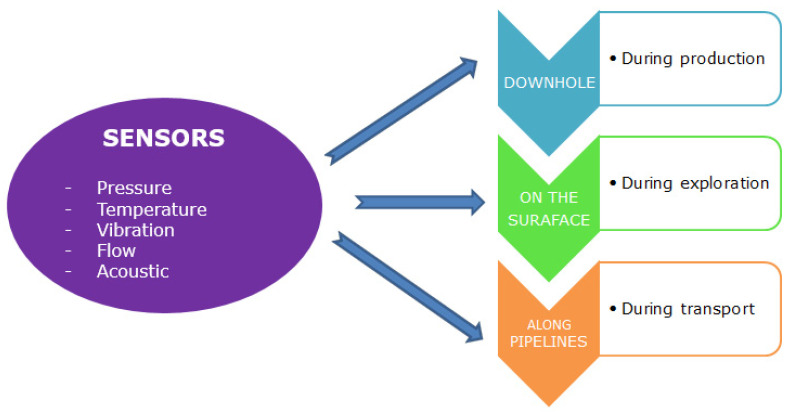
Sensor requirements in the O&G industry.

The goal of this review is to explore optical fibre-based sensing technologies that can significantly boost the performance and withstand extreme conditions prevalent in offshore O&G environments. Sensing in the O&G sector involves the measurement of different parameters like pressure, temperature, vibration, flow and acoustics. Sensing should be carried out throughout all stages of O&G production, which means that the sensor should be active on the surface, along the pipeline and even in the down-hole. The measurements are to be taken throughout the well, from the surface to the total depth of the oil well reservoir [2]. Figure 1 shows the various stages where sensors are required in the O&G industry.

The elemental part of optical sensor design is the identification of the key technology which best suits the needs of the O&G industry. There are different optical sensing methodologies, but the selection needs to be carried out considering the environmental conditions, application (downhole, on the surface or along the pipelines), level of sensitivity and accuracy required in physical parameter measurements.

With the worldwide decrease in oil reserves, exploitation of challenging reservoirs has rapidly started. In comparison to standard wells, the overall production of such reservoirs entails high and complex performance instrumentation, for example, fibre-optic distributed pressure, distributed strain monitoring, distributed temperature monitoring, etc. The development of fibre-optic sensory technology with regards to the oil and gas industry is currently on the rise and represents the future of well monitoring. Monitoring and data transmission via the utilization of fibre-optic sensors and standard optical fibre in cabling is currently common in the refining of natural gas and standard crude oil throughout the world. With the trend of hydrocarbon consumption outpacing its given discovery, techniques in Enhanced Oil Recovery (EOR) are being deployed worldwide so as to increase the apparent recoverable assets in the known reservoirs. Fibre-optic Monitoring represents an opportunity for the current oil and gas industry to manage and subsequently optimize its resources in a more effective manner and provide real-time data in a continuous way without interrupting production and reducing well intervention. Visiongain had forecasted that the expected expenditure on Fibre-Optic Monitoring by the gas and oil industry will be increasing globally [21]. The rise of relatively expensive multi- lateral hydraulic fracturing, the continued strength of the given capital expenditure for EOR and the intense focus on improving oil recovery make provision for the main markets for the uptake of Fibre-optic Monitoring over the next 10 years [21]. Although this exquisite technology has been around for a significant period, researchers are still investigating relatively new ways that this technology can withstand relatively higher temperatures and significant pressures with little disruption. Furthermore, the application opportunities within the current oil and gas industry for fibre-optic monitoring are poised to enable a considerable growth in spending on fibre-optic monitoring equipment. Increased digitization in the oil and gas industry has led to an increase in the use of fibre-optics sensing systems for production and pipeline monitoring. The global DAS market is projected to reach USD 792 million by 2025 from USD 513 million in 2019, which is considered to be an impact of COVID-19 [22].

This review paper mainly aims to cover sensors for oil and gas downhole applications. The following Section 2 will introduce basic elements of a fibre-optic sensor system, classification based on sensing methodologies (spectral-based, phase-based and polarisation-based) and their comparison. Section 3 describes different types of distributed fibre-optic sensors used in oil and gas applications and Section 4 further explains distributed temperature sensors (DTS) used for oil and gas well monitoring and their different configurations.

## 2. Optical Fibre-Based Sensors

An optical fibre sensor is composed of an optical fibre, transducer or a sensing element, detector and a light source [23]. Optical fibre is the medium through which light can propagate and the underlying principle is total internal reflection.

Fibre-optic sensing technology has several inherent advantages which makes them very attractive for a wide range of industrial sensing applications. The optical fibre is typically made up of a cylindrical waveguide that consists of a thin core with a refractive index covered by a cladding layer with a refractive index usually lower than that of the core for a single-mode fibre (SMF).

An SMF is made up of the core and cladding layers which are usually from fused silica. Light is propagated through the core of the fibre by total internal reflection (Snell’s law) [24] and to achieve a high refractive index, the core of the fibre is usually doped with germanium. To allow for the light energy to be maintained within the core, the refractive index of the core must be greater than that of the cladding. Multimode fibre has a larger core diameter that allows multiple modes of light to propagate. Photonic Crystal Fibres (PCFs), also known as microstructured fibres, represent another class of optical fibre that has a specialised geometrical structure (core-air hole cladding) and unique properties like guiding mechanisms and modal characteristics, making them an interesting candidate for a range of applications. To analyse polarisation effects, special fibres with modified core or cladding structures are needed. Polarisation fibres guide only one polarisation direction, thus polarising light is propagated through the fibre.

Standard telecom fibres are protected by coatings/buffer which protects the optical fibres from mechanical and environmental stresses. The primary coatings are applied on single or dual layers. The buffer material provides an additional layer of protection. For industrial applications like oil and gas, with harsh environments/HPHT (High-Pressure High Temperature) field conditions, special polymer coatings like polyamide [25,26,27] and acrylate [28] coatings are used which considerably increases the sensor sensitivities to temperature and strain. Standard coatings on the other side are not specified for environmental parameters, which can differ from the conditions in a downhole [29].

**Figure 2:**
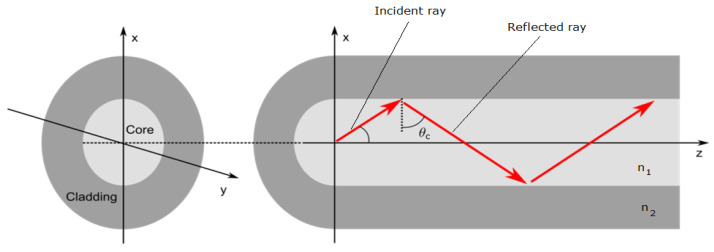
Schematic of light propagation through an optical fibre.

Figure 2 illustrates the light propagation through an optical fibre. If the angle of incidence of the incident ray is greater than the critical angle $\left(\theta_{c}\right)$ then the light ray gets reflected and confined within the core, else it is refracted. The critical angle is defined by Snell’s law and is given by:

$$\sin\theta_{c}=\frac{n_{2}}{n_{1}}$$

where $n_{1}$ and $n_{2}$ corresponds to the core and cladding refractive indices, respectively.

PCFs are a special class of optical fibres with a complex refractive index profile that employs a microstructured arrangement of low-index material in a background material of higher refractive index [30]. Normally the background material used in PCFs are pure or undoped silica and the low index cladding region consists of many air voids, also known as air holes [31]. Typically, photonic crystals are periodic optical (micro or nano) structures running axially along the length of the optical fibre, which affect the propagation characteristics of the electromagnetic waves travelling through its core [32].

**Figure 3:**
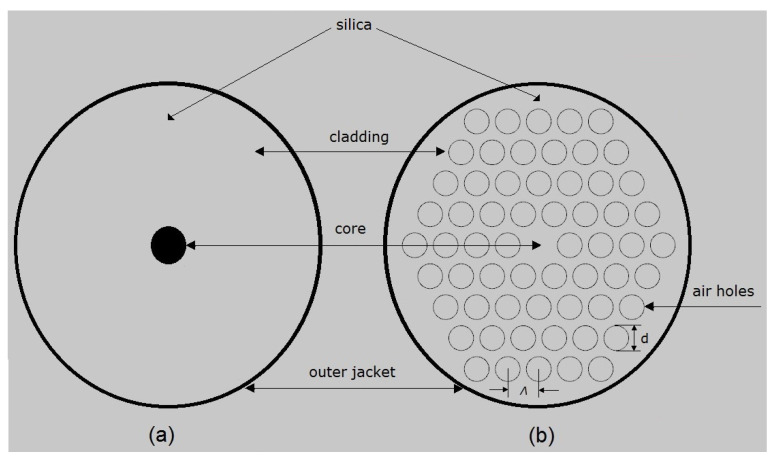
Cross-section of: (**a**) step-index SMF and (**b**) solid core PCF.

Figure 3a,b show the cross-sectional view of the structural difference between a standard step-index SMF and a solid core PCF with microstructured cladding. The geometrical parameters of PCF comprise: pitch (Λ) which is the cladding hole centre to centre distance and the diameter of the cladding air hole (d).

Optical fibres can be used as sensors for sensing various physical parameters like temperature, pressure, strain, etc., wherein the parameter to be sensed modulates various properties of light. From the basic principle of light propagation through an optical fibre, the light propagation changes when subjected to varying environmental conditions such as temperature and strain. By analysing the changes of the light properties through the fibre, the environmental conditions themselves can be determined. Fibre-optic sensors have been designed and developed to measure a wide range of physical parameters such as pressure [33], temperature [34], position [35], etc. Light propagated through an optical fibre can be characterized by parameters such as intensity, phase, wavelength and polarisation. The detection of the changes of these parameters as the optical fibre interacts with external perturbations can lead to the design of optical sensors capable of measuring a variety of physical parameters. As a result, fibre-optic sensors can be based on intensity measurement, phase (interferometric) measurement, spectral (wavelength), polarisation modulation and also the physical quantity they measure and their effect on the electric field of the optical signal. Existing optical fibre sensing technologies are categorised based on the effects used to measure physical phenomena and also the light modulation techniques used. In optical fibre sensors information is conveyed as a variation in intensity, frequency, phase, polarisation, wavelength or their combination of light [36]. Optical fibre sensing techniques like Raman [37], interferometry (Fabry-Perot, Michelson) [38,39,40], fibre Bragg gratings (FBG) [41], Brillouin [42], etc., are proficient to monitor different physical parameters such as pressure, temperature, strain, chemical concentration, flow, etc. Extensive research into the advancement of optical fibre technology for variety of applications have been ongoing for the past 30 years which have laid the technical background for the various categories and their applications are expanding rapidly.

Fibre-optic sensors are further divided into two subcategories: intrinsic and extrinsic sensors. For intrinsic sensors, the light is confined within the optical fibre in which the physical quantity acts on. The performance of intrinsic sensors is largely dependent on fibre materials. While for extrinsic sensors, the light exits the fibre, get modulated by the external perturbation and is relaunched back into the fibre. The performance, in this case, is largely independent of the fibre material but on the sensing element.

**Figure 4:**
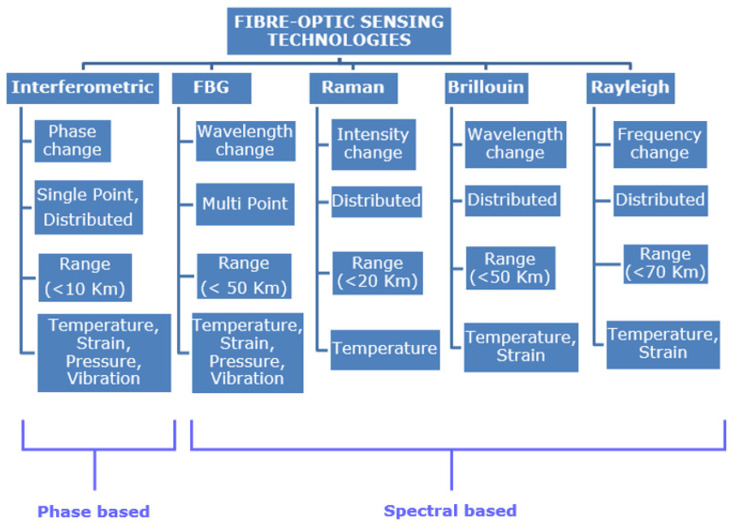
Comparison of fibre-optic sensing technologies.

**Figure 5:**
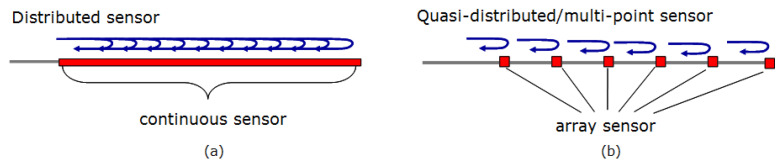
(**a**) Distributed fibre-optic sensor and (**b**) quasi-distributed fibre-optic sensor.

Figure 4 shows the comparison between different optical fibre sensing technologies [38,39,40,41,42,43,44]. The various sensors compared in Figure 4 are all intrinsic type fibre-optic sensors. Figure 4 compares the most widely used fibre-optics sensors, however, there are other categories like polarisation-based sensors, Distributed Chemical Sensors. Distributed fibre-optic sensor (Figure 5a) enables continuous measurements along the entire length of the sensing fibre, whereas quasi distributed or multi-point sensors (Figure 5b) carries out sensing along specific points of the fibre sensors. Multimodal fibre-optic sensors are capable of sensing multiple sensing modalities like phase, wavelength, polarisation, etc., which can be utilised for multi-parameter sensing. Multi-parameter sensing involves sensing multiple parameters, which can be physical parameters like temperature, pressure, vibration, etc., or chemical parameters.

Propagation of light through the fibre is usually used to determine measurement information by spectral characterisation (wavelength/frequency and intensity), phase characterisation (interferometry) and polarisation. In the following sections, operation principles based on various techniques stated above are reviewed in detail.

### 2.1. Wavelength Modulation-Based Sensing

Wavelength modulation-based optical sensing is one of the spectral techniques which measures physical parameters by detecting a change in wavelength when the optical fibre interacts with the measurand. The most common type of wavelength modulated optical sensing is fibre grating-based sensing. A grating can be produced when the core of the fibre is exposed to an intense UV laser light and was first discovered in 1978 by Hill [45]. The fibre Bragg grating sensors and long periodic grating sensors have since been developed based on this technique and have found many applications in measuring temperature, pressure and strain [46]. Fibre Bragg grating-based sensor was developed to measure hydrostatic pressure with a typical resolution of 0.5% by specially coating the grating region with different materials [28,47,48]. A side hole fibre Bragg grating (FBG)-based pressure sensor offers superior pressure sensitivity and lower temperature sensitivity allowing straightforward temperature compensation techniques to be used to form a practical downhole distributed pressure measurement system [49]. The reflectivity from the FBG sensor can be enhanced by optimising the grating parameters like grating length, effective refractive index and grating strength which is beneficial for long-distance oil and gas remote sensing applications [50]. The multiplexing capability and the zero optical power loss of the fibre grating-based sensors have led to more research in this technology. However, the long term stability and reliability of this technology have been a major challenge due to the degradation of its mechanical strength and optical properties when exposed to harsh environments [51]. Additionally, despite the many opportunities these sensors have been slow in replacing the conventional electronic sensors over other technologies, many concerns still exist. The issue of cross-sensitivity limits the scale of this technology when used in harsh environments. For fibre grating-based sensors to be used in real applications, these issues and challenges have to be minimised. FBG sensors are described more in detail in Section 2.5.

### 2.2. Intensity Modulation-Based Sensing

**Figure 6:**
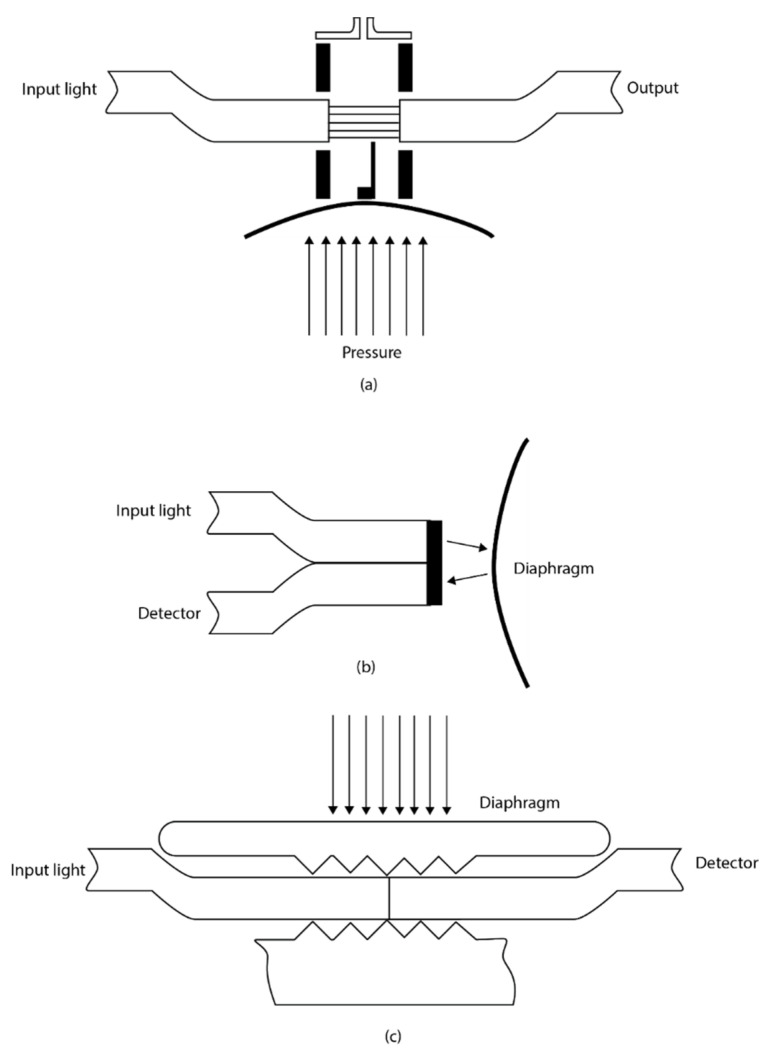
Intensity modulated-based sensing class; (**a**) Transmission intensity (**b**) Reflection intensity (**c**) microbending intensity type [52].

Intensity modulation-based sensing is another spectral technique that measures physical quantities based on the principle of direct detection of the change in optical power in either reflection or transmission. They are inherently simple devices where light from an optical source is propagated through the fibre and the intensity is altered at the transducer which is then returned to an optical detector. The light intensity detected by the detector is a function of the physical quantity measured. There are three different classes of intensity modulation-based sensing as shown in Figure 6. They are transmission intensity, reflection intensity and micro bending intensity.

The transmission coupling-based sensor consists of two fibres with a small gap between them, wherein the amount of light coupled to the second fibre depends on the fibre acceptance angle and the distance between the fibres. One of the fibres can move in response to vibration or pressure thereby changing the distance between the fibres and hence the coupling loss [53]. The reflection-based sensor operates in a similar fashion, where light is reflected from a flexible diaphragm back into a collecting fibre. The reflected light intensity changes as the diaphragm is flexed. Once the coupling relationship between the input fibre, diaphragm and collecting fibre is known, intensity changes can be related to the applied displacement or pressure [53]. These fibre-optic sensors can effectively measure displacement or dynamic pressure.

Intensity modulation-based sensors made with multimode fibre-optic micro bend have successfully been commercialized. It is based on the operating principle in which the mechanical periodic micro bend coupled the energy of both the radiation and guided modes thereby resulting in the attenuation of the transmitted light. Sensor configuration can be designed and constructed in such a way that the mechanical mirobending device transfers the applied perturbation to the optical intensity change. The micro bend modulated sensors have been reported to have good performance characteristics like good resolution [54], however, the fluctuation of the light source and large hysteresis posed a limit to their accuracy [55]. Moreover, the large size of the microbending device makes it very difficult to be applied in many sensing applications. Raman distributed temperature sensors (DTS) are based on intensity modulation techniques and can be efficiently used for real-time downhole monitoring [56]. Raman DTS is described in the upcoming Section 2.6.

### 2.3. Interferometry-Based Sensing

**Figure 7:**
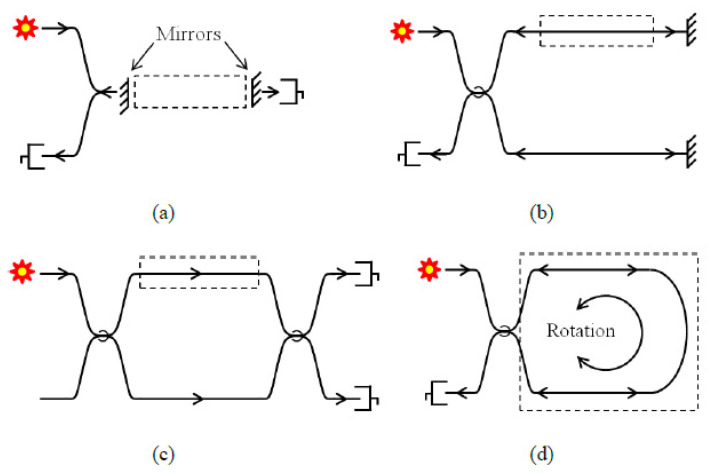
Phase modulation-based sensing uses the interferometric techniques (**a**) Fabry–Perot interferometer (**b**) Michelson interferometer (**c**) Mach–Zehnder interferometer and (**d**) Sagnac interferometer [52].

Phase modulation-based sensing uses interferometric mechanisms such as the Mach–Zehnder, Michelson, Sagnac and Fabry–Perot to measure physical parameters see Figure 7. It works based on the principle of constructive interference by observing the change in interference between two light beams. Interferometric modulation-based sensing has become a very useful tool for high precision sensing, optical spectrum analysis, construction of lasers and optical wavelength filtering [57]. This sensing technique uses the interference between two parallel beams propagated through different optical paths or fibre. It relies on the change in the cavity length of one of the interference arms. The cavity length change can be as a result of either the refractive index change or the change in fibre length. The different types of interferometric-based sensing have slightly varying sensing mechanisms.

**Figure 8:**
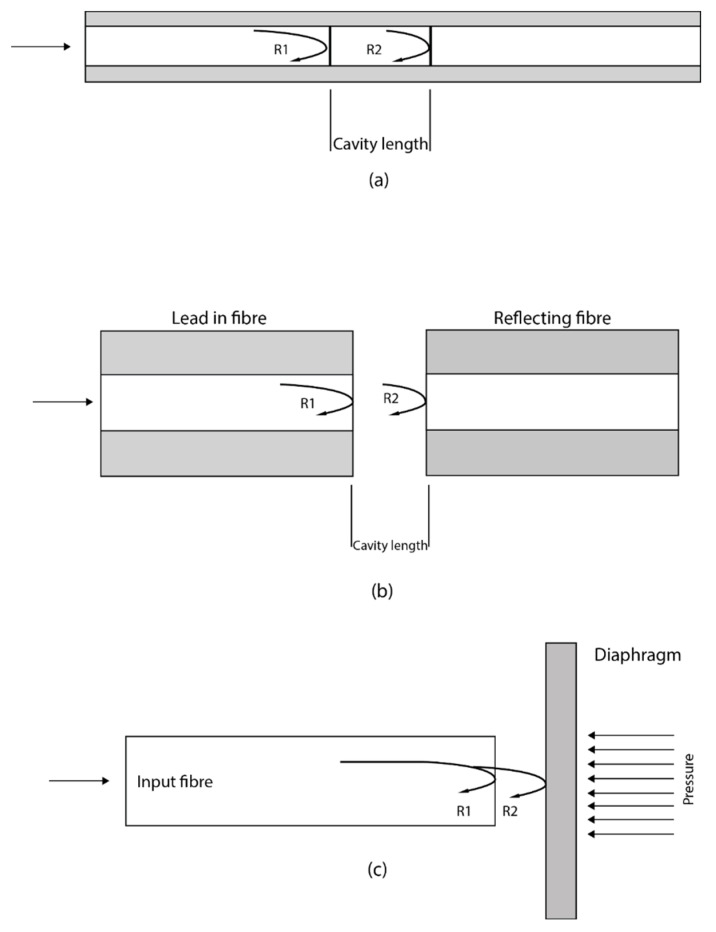
Different Fabry–Perot configurations (**a**) Illustration of IFPI formed with a single optical fibre (**b**) Arrangement of an EFPI formed using two optical fibres and (**c**) Schematic of an FP cavity formed using an optical fibre as the lead-in fibre and a deformable diaphragm [52].

Fabry–Perot interferometer is generally designed to measure physical parameters through the formation of a cavity with two parallel reflective surfaces. When light is propagated through the Fabry–Perot cavity, multiple interferences of light are formed caused by the multiple reflections between the two reflective surfaces. The Fabry–Perot interferometer is subdivided into two categories, intrinsic Fabry–Perot interferometer (IFPI) and extrinsic Fabry–Perot interferometer (EFPI). In IFPI, the light is confined and modulated within the fibre. The cavity length and modulation are from within the confine of the fibre. In EFPI, the light exits the fibre and is modulated before being relaunched back into the fibre. The cavity length is formed outside of the fibre and the fibre serves as the medium for transmitting light into and out of the Fabry–Perot cavity. Different configurations for FP have been proposed and developed. One configuration is to place a different fibre aligned to the first one which forms a cavity between them and is then packaged using a glass or silica, see Figure 8. An alternative method is the use of a diaphragm at the opposite side of the fibre end forming a cavity. Any change in the cavity length due to the deformation of the diaphragm would result in changes in interference [58].

The IFPI is usually fabricated by splicing a special fibre and coating the two end-faces with a reflective film. The output signal is generated by the superposition of the multiple reflections at the end-faces of the special fibre. These reflections are a result of the reflectance of the coating, the cavity and the refractive index of the fibre. Whenever there is a change in the cavity length or the refractive index, the interference output can be tracked thereby being able to measure any physical quantity that caused the changes of the optical properties [59].

In EFPI, the performance of the sensor depends only on the sensing element which gives it the flexibility to adapt to various sensing applications. An EFPI cavity is formed by placing an input fibre side by side to a reflecting fibre. As light is propagated through the input fibre, a fraction of it is reflected back as R1, approximately 4% and all others are transmitted into the cavity space to the reflected fibre end-face. Part of the transmitted light is also reflected back as R2 at the end-face of the reflected fibre which is then recoupled into the input fibre. Fibre-optic interferometric acoustic sensor array has established itself as a potential alternative to the conventional sonar array based on electro-ceramic transducers for underwater or subsea applications [59]. A metal-coated hybrid sensing system based on FBG and Extrinsic Fabry–Perot Interferometer (EFPI) cavity for high-pressure high temperature (HPHT) measurement has been reported for subsea underwater applications wherein the FBG and EFPI are used to measure temperature and pressure, respectively [60].

EFPI sensors have been developed and commercially available. The EFPI sensors have many advantages over the Mach–Zehnder and Michelson sensors such as their high sensitivity, small size, good flexibility, and their simple structures. These make them very attractive for various sensing applications. However, the EFPI have the potential of having low coupling efficiency due to the usual misalignment of the reflecting fibre.

Liao et al. reported a high temperature (Up to 950 ºC) sensor based on micro taper in-line fibre Mach–Zehnder interferometer, which has got wide application prospect in the fields of high-temperature hot gas flow, as well as oil and gas field development [61]. Zhao et al. introduced an ultrasensitive temperature sensor with Vernier effect-improved fibre Michelson interferometer which is suitable for various applications that need high precision temperature measurement [62]. A sensitive fibre-optic vibration sensor based on mixed Sagnac/Mach–Zehnder interferometers has been reported for urban gas pipeline leak detection [63].

### 2.4. Polarisation Modulation-Based Sensing

Polarisation-based fibre-optic sensors typically involve an extrinsic birefringent component to perform the actual polarisation modulation [64]. These sensors are designed in such a way that the applied pressure changes the polarisation properties of the medium or the optical fibre due to the photo-elastic effect [65]. While the sensors based on Faraday’s effect measure both electric and magnetic fields. Measurements based on Faraday’s effect have found applications in electric current measurements. Optical electric current sensors find application in electric motors used to drive subsea valves and chokes [66]. Magnetic sensors play a critical role in drilling wells into a target reservoir zone by providing directional data of the well and acquiring information about the surrounding geological formations [67].

Optical sensors based on the photo-elastic effect was first introduced in 1982 by Spillman [68]. After then, many sensing applications based on the photo-elastic effect have been developed and reported to help solve the challenge of compensation for the power variation [69]. Silica and glass fibres show a weak photo-elastic effect; however, external crystals are used as better sensing elements for more accurate measurements.

### 2.5. Fibre Bragg Grating Sensors

**Figure 9:**
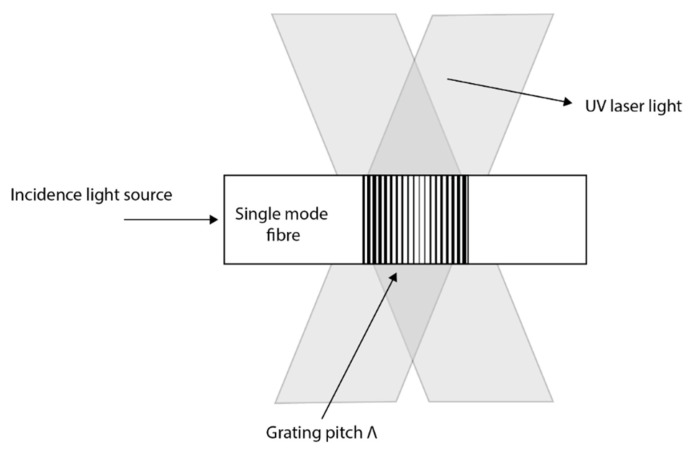
Refractive index change of fibre Bragg grating [52].

Fibre Bragg grating can be produced from a standard single-mode optical fibre. The core of the fibre is exposed to two ultra-violet light beams originating from the same laser source, the UV light beams constructively and destructively interfere (Figure 9). The result is a grating recorded into the fibre as a periodic variation of the refractive index of the fibre core. When light is propagated into the fibre, a narrow waveband of light is reflected back at the grating while other wavelengths of light are transmitted. The wavelength of the reflected light varies with the period of the grating which itself varies with both strain and temperature. The central wavelength of the reflected signal is generally called the Bragg wavelength ($\lambda_{B}$) and it has a linear relationship between the refractive index and the period of the grating $\lambda_{B}=2n_{eff}\Lambda$. This means that any variation in strain and temperature to which the optical fibre is subjected can cause a shift in the Bragg wavelength. The effective refractive index is $n_{eff}$ is a physical characteristic of the fibre-optic material in which the grating is formed, while the grating period Λ depends on the design of the grating. The changes in temperature and applied strain on the grating of the fibre directly modify the refractive index or the grating period of the fibre, respectively [52].

**Figure 10:**
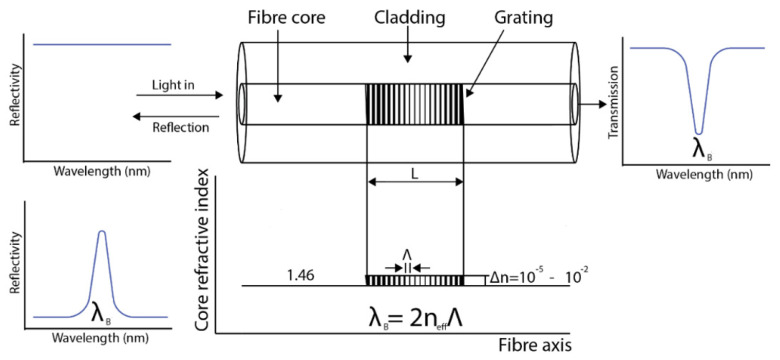
Schematic of FBG and its reflection and transmission properties [52].

**Figure 11:**
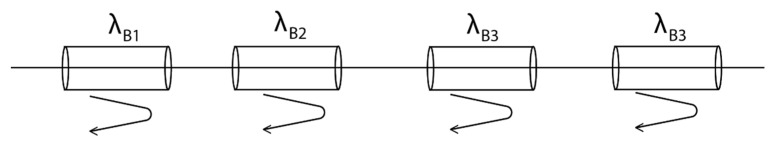
Multipoint distributed (WDM) pointing sensing based on FBG [52].

Figure 9 shows how an FBG is written within the core of a single-mode fibre. Figure 10 shows the schematic of FBG and its reflection and transmission properties. These simple principles have made it possible for various advancements of the FBG sensing technology and also take advantage of the multiplexing capability of FBG. See Figure 11 for a multipoint distributed FBG sensing configuration. A corrosion-resistant FBG-based quasi-distributed sensor has been reported for crude oil tank dynamic temperature profile monitoring [47,48,49,50,51,52,53,54,55,56,57,58,59].

FBG sensor is primarily composed of three main segments; the sensing part, which is composed of the bare FBG that does the actual sensing, the packaging and the FBG arrays; the instrumentation which is composed of the interrogating instruments and related components such as the switches, multiplexors, data acquisition system, data processing units, software and the graphical user interface; and the system integration unit which compose of project management and engineering aspects [70].

Hill et al. observed photosensitivity of the optical fibre when exposing the germanium doped fibre core to the two coherent argon-ion laser counter-propagating radiation with 488 nm wavelengths [45]. The result showed a periodic change in the refractive index similar to the periodic pattern of the interference wave of the laser. Both the reflected light from the grating and the writing laser have the same wavelengths. After about 10 years later, Meltz et al. [71] in 1989 presented a holographic technique to address that limitation by using a writing wavelength of 244 nm (5 eV) that made it possible to write gratings with wavelength other than the wavelength of the writing laser. The couple mode theory [72] have been developed to analyse the spectral response of the fibre Brag grating by Erdogan et al. [73]. Lemaire in 1993 [74] proposed the use of the Hydrogen loading technique to improve the optical fibre photosensitivity prior to laser irradiation. In 1993, the phase mask technique [75] was developed that successfully superseded the success of the transverse holographic method by Meltz. In 1995, Othonos [76] demonstrated an improvement in the writings by relaxing the need for close contact. Femtosecond-laser-inscribed FBGs are promising for industrial applications such as nuclear plants, oil and gas explorations with harsh environmental conditions. This is because femtosecond laser pulses can induce effective refractive index modulation in almost all types of optical materials, including non-photosensitive optical fibres [77]. Femtosecond laser phase mask technology, femtosecond laser holographic interferometry, and femtosecond laser direct writing technology are the three fabrication technologies developed for fabricating FBGs in various fibre types. The direct writing technology includes point-by-point (PbP) inscription method and line-by-line (LbL) scanning method [77]. Qiao et al. developed FBGs with high temperature resistance of up to 1100 °C using femtosecond laser side-illumination and experimentally realized high-temperature and pressure measurements up to 400 °C and 100 Mpa [78]. The long-term stability and repeatability of downhole sensors also depends on the packaging techniques and not just the FBG performances. Qiao et al. has introduced an alloy material of Nb-40 Ti-5.5 Al for FBG packaging which serves as a metal transducer [78].

Recently, many fibre-optic sensors are made with FBG through the modulation of the refractive index profiles. Different types of FBG have been reported based on their coupling characteristics. First, there is short-period grating FBG [45]. Here, the grating period is typically within the range of 0.22–0.54 μm with the light coupled into the backward propagation direction. Second, tilted period grating [79,80]. In this short period of grating, the grating is tilted by an angle with respect to the axis of the fibre. The light with the tilted grating can also be coupled into the backward propagation direction. Lastly, the long period grating [81,82]. The grating period of the long period grating has a length within the range of 100–500 μm with the light coupled into the forward propagation direction. They act as loss filters and are usually used as a gain equalizer. They have been successfully manufactured by exposing the core of the fibre to point by point UV light. Other examples include chirped FBG, apodised FBG and phase shift FBG [83,84,85].

### 2.6. Distributed Fibre-Optic Sensing (DFOS) Technologies Based on Rayleigh, Raman and Brillouin Scattering

Distributed sensing uses scattered light to detect, measure and analyse certain backscattered characteristics and changes that occur in the optical fibre. Rayleigh, Raman and Brillouin type scattering are used in order to measure those changes. Distributed systems are based on two main techniques: optical time-domain reflectometry (OTDR) and optical frequency domain reflectometry (OFDR) with the purpose of measuring the optical loss distribution along a fibre in order to evaluate along the fibre its condition and to detect, locate and quantify the losses and defects all along. In this principle, a pulse of light is launched through the fibre and the optical properties of the backscattered light are measured [86].

**Figure 12:**
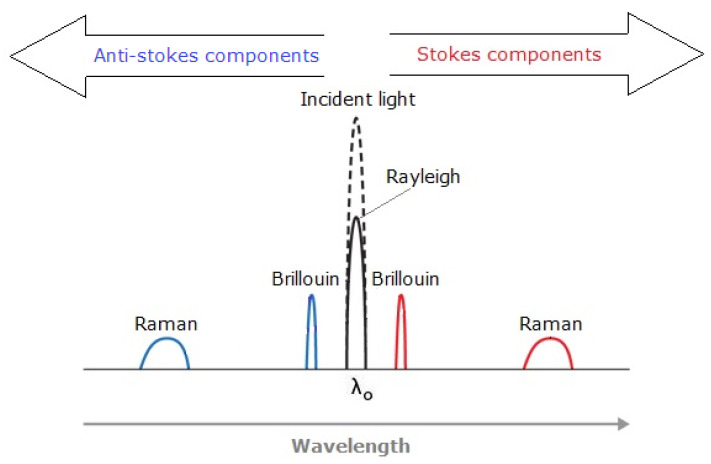
Backscattered spectrum with Rayleigh, Brillouin and Raman bands, as well as the stokes and anti-stokes bands.

**Figure 13:**
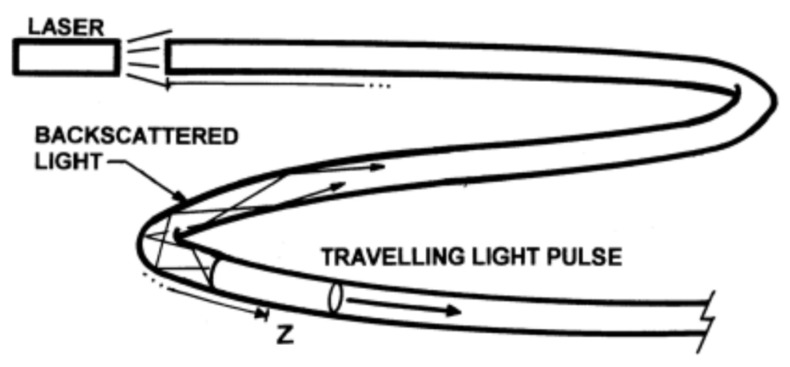
Travelling light pulse sending backscattered light back to the instrument box [86].

There are three main principles for distributed sensing, the backscattered light contains three spectral parts: Rayleigh, Raman and Brillouin as shown in Figure 12. All the distributed sensing technologies are similar, a laser light pulse is sent down the fibre and some of the light is scattered back (backscattered light) by the Rayleigh, Brillouin and Raman effects (Figure 13). The backscattered spectrum is monitored as the light pulse propagates through the fibre which can be correlated to the changes in the fibre (due to temperature, pressure, etc.) at various locations.

Raman scattering contains information on the temperature to be able to calculate the temperature along the fibre. It is composed of the Anti-Stokes band (strong dependent on temperature) and the Stokes, which can give temperature with high precision at the location that the scattering is taking place. It is only temperature-dependent.

Rayleigh scattering effect is elastic and is used for both temperature and strain monitoring. There are no gains or loss of energy meaning that occurs at the same wavelength as the light signal.

The main characteristics of this system are a high resolution of measured parameters and short spatial resolution, but the maximal length of the sensor is limited to 70m. Therefore, this system is suitable for monitoring localized strain changes over relatively short distances [87]. Most distributed acoustic sensing (DAS) systems, where the signal-to-noise ratio (SNR) is very important, are based on Rayleigh scattering.

Brillouin scattering is also temperature- and strain-dependent. The wavelengths of the Brillouin, Stokes and Anti-Stokes peaks occur close to the input signal, so sophisticated filtering methods are needed to filter the Rayleigh signal and improve SNR. Since Brillouin scattering is both temperature- and strain-dependent, most DTSS (Distributed Temperature and/or Strain Sensing) systems are based on Brillouin scattering [88]. Various distributed fibre-optic sensors such as DTS (Distributed Temperature Sensor), DAS (Distributed Acoustic Sensor), DTSS ((Distributed Temperature and/or Strain Sensor), DPS (Distributed Pressure Sensor) and DCS (Distributed Chemical Sensor) used in oil and gas industry are explained in the following section.

## 3. DFOS in the Oil and Gas Industry

### 3.1. Distributed Temperature Sensing (DTS)

The DTS use the OTDR operating model based on Raman scattering in the optical fibre [89], which transduces temperature into an optical signal. The DTS box beams laser lights along the optical fibre sensor in pulses. The travelling light pulses experience scattering with some of the lights directed back to the DTS box, Raman backscattered light, which comprises two bands: anti-Stokes and Stokes. The intensity of the anti-Stokes band changes with temperature while the other remains constant. By calculating the intensity of the ratio of the anti-Stokes backscatter and the Stokes backscatter we are able to obtain the temperature in the fibre. The temperatures are not recorded at points, but they are recorded along the cable that serves as the optical sensors. They can measure temperatures of up to 300 degrees Celsius per meter [90]. Using conventional OTDRs, a range of approximately 15 kilometres can be obtained for the DTS [91]. The DTS resolves both its temporal and spatial temperatures at 0.1 degrees Celsius. It has also a spatial resolution of 1.5 m [89].

Typical well monitoring applications of the DTS include flow metering, flow assurances, leak detection, gas-lift surveillance, and permanent temperature logging [70,89]. DTS for Oil and Gas Well Monitoring and its various configurations are explained in detail in Section 4.

### 3.2. Distributed Acoustic Sensing (DAS)

The DAS uses optical fibre for sensing and telemetry. The DAS is an OTDR-based system that uses Rayleigh backscatters in a single-mode optical fibre to measure sound frequencies over large distances and in harsh environments. It is mostly used in the sensing of strains in well systems. Using an acoustic-based system allows the operators to reconstruct the source event and subject it to real-time analysis. The optical pulse that is propagating along the fibre experiences attenuation along the optical fibre. The acoustic signals obtained from each position along the fibre from the processed backscattered light are then analysed and sent to a display [92].

The DAS system is ideal for the detection of relative strains wherein the operators use the optical fibre’s index of refraction and speed of light to determine the distance along the fibre that is experiencing the strain. However, the accuracy of the measurement is dependent on the distance that the light probe travels. For this reason, the DAS system pulses the light strobe a thousand times per second to obtain a high signal to noise ratio [93]. The carrier level is determined by the signal amplitude while the noise is determined by combining different sources including the detector, electronic components, and laser light pulses. The maximum operational range of the DAS is limited when the pulse’s amplitude becomes very low to the extent that it cannot be able to send a clear signal. An increase in the power of the input signal cannot remedy this decline of the maximum range. Exceeding the recommended maximum range will result in non-linear optical effects that are eventually disruptive to the entire optical fibre system. The DAS system’s effective fibre length is 10 km. The spatial down sampling is equal or less than 1 m while the frequency range is up to 10 kHz [94].

DAS is preferable in wellbore diagnostics to monitor various factors. It can be used to determine the location of a leak flow caused by pressure bleeding. It can differentiate a single-phase flow from a two-phase flow. The peak noise can estimate the rate of leaking. The system can also distinguish between the matrix flow, borehole flow, fracture flow, and channelling [93]. The appropriateness of the DAS system in these operations is due to the following factors. First, the low acoustic frequencies enable the DAS system to provide a continuous full wellbore coverage [95]. Second, the acquisition of operational data from an entire well takes only minutes. Third, the costs of deployment are low. Fourth, the DAS system is safer because one is not required to rig up for logging.

### 3.3. Distributed Temperature and/or Strain Sensing (DTSS)

The DTSS is based on the Brillouin scattering mechanism that employs the OTDR system. The system analyses both the thermal and strain effects of the target object by combining both the DTS and the DSS system. The DTS measures temperature properties while the DSS provides measurements that are used to the location and severity of deformation in well casing. The DSS also provides insightful data that estimate the stresses inherent at perforations during oil output stimulations. Most DTSS systems are based on the technology that combines the Brillouin Optical Time Domain Reflectometer (BOTDR) with the Brillouin optical time-domain analysis (BOTDA). The BOTDA preceded the BOTDR in sensor detection. It used two lasers that counter-propagate by exploiting the benefits of Brillouin amplification. However, the system was limited to a temperature accuracy of 3 degrees Celsius and a spatial resolution of 100 m over a sensing length of 1.2 km. The BOTDR was introduced because it could monitor the system from the opposite end [96]. As a result, the 1.2 km limitation in the temperature measurement range was increased to approximately 11 km without a change in the temperature accuracy or spatial resolution. An optimally tuned DTSS system can have a maximum temperature measurement range of over 50 km.

The DTSS system is ideal because it can use loop or single end measurements [97]. For that reason, the failure of the BOTDA part of the system is not a worry because the system switches to the more stable BOTDR system. Second, the system is affordable because it uses a low-cost telecoms fibre that is capable of detecting both temperature and strain. Third, the system has a high spatial resolution and range. Fourth, the combination of the BOTDA and the BOTDR system enables the operator to monitor a well using multiple channels. These features make the DTSS system ideal for the monitoring of a leakage in a well for distances of up to 140 km.

### 3.4. Distributed Pressure Sensing (DPS)

The core principle of DPS is the conversion of hydrostatic pressure acting along a coated optical fibre, into a distributed mechanical strain [98]. Measurements of distributed pressure can be thus inferred by converting the applied hydrostatic pressure into distributed mechanical strain acting on the fibre, and measuring the strain changes by the Brillouin scattering frequency shifts they experience [99].

The DPS is preferable because it does not require expensive optical devices. A light source and a light detector are the main elements that are needed in this technology. However, the DPS has some inefficiencies based on the intrinsic nature of the measurements. For example, the intensity of light often depends on quite a number of factors that cannot be controlled easily or cannot be controlled at all. The light source’s intensity of light is often subject to fluctuations because of certain factors such as temperature changes and ageing [100]. Oxidation can also affect the efficiency of the reflectivity of the diaphragm mirror. The aspect of fibre bending with the objective of increasing power input can also affect the intensity of the light that is being emitted. This disadvantage is always corrected by fixing the pathway of the fibre-optics. However, compensations are always made while calibrating the pressure sensor system. It is important to note that these provisions meant to accommodate the errors mentioned above are subject to bias and can question the accuracy of the system. Zhang et al. reported an ultra-high sensitivity distributed pressure sensor with a sensing range of 1.05 km and a spatial resolution of 5 cm [101].

### 3.5. Distributed Chemical Sensing (DCS)

Chemical sensors in fibre-optics operate in a manner that allows the transportation of the light by intensity or wavelength in order to provide vital information concerning the analytes in the immediate environment that surrounds the sensor [102].

A distributed chemical sensor is normally developed by coating multiple Bragg gratings in a single glass fibre with chemical responsive coatings. In this configuration, FBGs are quasi-distributed or distributed point sensors.

A chemically selective layer is used to replace a part of the fibre’s cladding. The layer detects the environment in terms of light polarisation, reflectance, and absorbance changes. The light or propagation characteristics of the optical fibre measure the changes in the target object. Typically, the DCS is vital in the detection of formation water, enhanced oil recovery water breakthrough, H_2_S and the CO_2_ in carbon capture reservoirs and approximating the identity of specific molecules in diverse media and under extreme conditions. A DCS modified by the coated fibre Bragg grating will produce an axial strain in the fibre in the presence of a chemical compound [94]. The measurement of the different chemical compositions is feasible if different coatings are applied on the FBG coats. This coating has a reversible absorption property because it repels water and absorbs the target analyte. The presence of a chemical compound will induce a wavelength shift of the FBG.

However, this shift is only useful in determining the quantitative properties of the chemical compound. The accuracy of the results is also prone to external factors such as coating sensitivity, methods of processing the coating to the FBG, and the approach used to optimize the chemical selectivity of the polymeric coatings. For that reason, the selective measurement of a particular chemical compound will require the operator to tune the polymer coating on the FBG to suit the analytes [103].

## 4. DTS for Oil and Gas Well Monitoring

In recent times extracting oil and gas from reservoirs comes with unique challenges, increase in demand for energy with a shortage of oil produced has driven the petroleum industry to search for oil in more complex reservoirs. Due to the complexity of these reservoirs, the strategy for oil production has become more difficult and often economically impossible utilizing available technology. Reliable and efficient data are required for making decisions to combat the issues with uncertainty. Fibre-optic DTS based on the principle of Raman backscattering is more efficient for oil and gas well monitoring than conventional electronic sensors. However, one critical issue associated with the DTS system is the complexity in the intensity of the backscattering profile; this is as a result of local attenuation affected by physical perturbation as well as pure temperature effect [104]. Differential attenuation (DA) in optical fibre is the gradual loss of backscattered light intensity as it moves through the fibre back to a detector system. To achieve accurate temperature measurement, the DA in the optical fibre must be resolved by correcting this error in the DTS system. The attenuation error in a fibre-optic cable is due to the difference in wavelength of Stokes and anti-Stokes component which again depends on the laser pulse [86].

Fibre cable produced by manufacturers has different attenuation rates; attenuation can be enhanced during installation or thereafter by bending or twisting of the fibre cable, tension and chemical ingression of hydrogen gas which is very common in the oil and gas industry [84,105]. These conditions result in non-linear attenuation along the fibre cable which causes error in measurement when a single-ended configuration is applied [105]. A double-ended configuration of fibre cable addresses the issues with nonlinear attenuation by looping the cable across an area of consideration and connecting both ends of the loop to a DTS system. The result of the experiment conducted showed similar accuracy when compared with single-ended configuration with linear attenuation. However, twice the length of fibre cable is needed for this system (as compared to the single-ended configuration) when applied in an oil or gas well [105].

Furthermore, in the event of hydrogen darkening optical transmission capability of the fibre is limited. Space limitation can also pose a huge problem. Hence this method cannot be applied in all wells. Increased noise in temperature trace is prominent in the double-ended system due to the long length of fibre cable and consequently requires more time for data acquisition during measurement to reduce the noise level.

Another typical way in which this problem could be resolved is by sectioning out areas in the optical fibre cable known to have nonlinear attenuation and applying the Differential Attenuation Factor (DAF) for each section [106]. The issue with this method is that fibre condition changes over time and the attenuation rate of these sections will change as well hence it will require sectioning the fibre and making a correction on the DAF constantly.

This method may not be practical in cases where the attenuation rate changes continuously in wells. In instances where the fibre is installed in a well, acquiring the correct value for DAF will be difficult or impossible in most cases because the temperature at the end point of the fibre is required to obtain the DAF using this method [86].

The possible alternative to resolving these issues is the deployment of a single-ended optical cable configuration which can achieve the same accuracy as the double-ended configuration. DTS system works with the principle of Raman scattering for temperature measurement. The Raman signal has two bands, namely Stokes and anti-Stokes bands. To carry out this correction using a single-ended system, an extra light source is needed with a wavelength chosen in which the Stokes wavelength of the secondary sources (correction light source) coincides with the backscattered anti-Stokes wavelength of the primary source (measurement light source). The Stokes and anti-Stokes signals filtered from the two light sources produce the same attenuation rate during backscattering. This technique cancels out the DA automatically ensuring temperature measurements are accurate.

### 4.1. DTS Fibre Configuration in Oil Well

Fibre cable can be set up in oil and gas wells using three methods of configurations: single-ended, duplexed single-ended and double-ended configuration [105]. The method employed in the field can be a factor of cost, quality of information required and objective of installation.

All the configuration method applies the same principle of acquiring temperature data. The respective power level of the anti-Stokes and Stokes signals Stokes ratio) at each point of measurement along the length of the fibre in the well are compiled by the DTS system to generate the distributed temperature measurement. Temperature change across the fibre length at any given point will affect the Stokes ratio at the location of that point. The Stokes ratios of the entire length of the fibre are collected and interpreted by the DTS system [107]. Misleading values may be obtained due to the attenuation property of an optical fibre cable.

#### 4.1.1. Single-Ended Configuration

The single-ended installation is a straightforward system configuration of the fibre-optic cable in the well with one end connected to the DTS instrument and the other end ran into the well for measurement over the area of consideration. This system assumes uniform differential attenuation along the entire length of the cable for calibration purposes. However, damage in the fibre cable or connector points and fusion splices can affect the attenuation rate; this phenomenon is known as step losses [108]. This type of system configuration cannot be calibrated accurately which means that the absolute temperature cannot be known although its spatial resolution (ability to spot a small change in temperature) is high as any other configuration method. This method can be applied in well operations where the objective is to assess the shape of the temperature profile and to qualitatively infer down-hole flowing conditions with accurate calibration not essential [86]. This is applicable in cases when the same instrument box and fibre line are used in subsequent measurements. In cases where the attenuation rate changes in the fibre line or the instrument box are replaced, the measurement made will be erroneous except where proper calibration is carried out again.

In a well that requires DTS run over a long period of time, there is a great chance that different fibre or instrument boxes may be used over time, it is then essential for accurate calibration of the fibre cable to compare data. The temperature must be known down-hole to obtain accurate calibration for this configuration, a separate wire (electrical or fibre Bragg grating) can be run in the same capillary line to take measurement of the temperature at the end of the fibre line [86].

**Figure 14:**
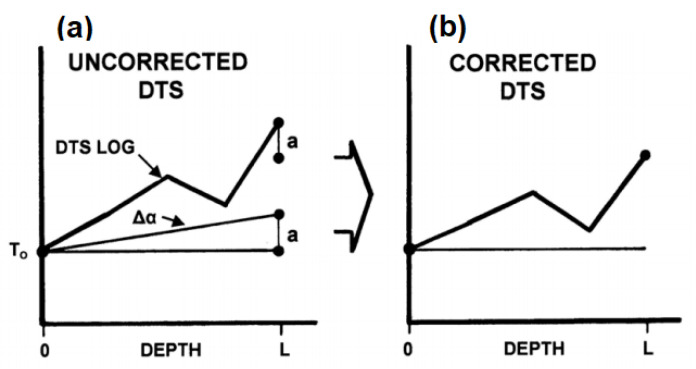
Constant differential attenuation representation with a temperature gauge for calibration [86].

Figure 14 shows a representation of a constant differential attenuation. The black dot in Figure 14 represents the temperature gauge in the well. The depths of the well range from z = 0 to z = L. The assumption of constant attenuation will not satisfy the case when the fibre cable has been in the well for a long time or exposed to water or hydrogen. From measurement at depth L by the DTS system when compared to the temperature gauge at the same depth, the temperature value from the temperature gauge is lower than the DTS log by the amount “a” which is a result of the differential attenuation error in the fibre.

If the temperature is known at the end of the fibre cable, the non-linear differential attenuation rate can be resolved by sectioning areas of the fibre with different attenuation factors and applying correction on the DTS system [86]. However, fibre conditions over time in many applications especially in an oil well tends to affect the transmission properties resulting in constantly correcting DA error over time which is not reliable.

#### 4.1.2. Duplexed Single-Ended Configuration

Partially returned fibre is also another installation method. It is pumped down to the required depth and returned partially back up the hole through turnaround subs. To terminate fibre movement at the predestined retuned depth, a valve is set at that point. It is expected in such a method that symmetry is exhibited about the turnaround sub for both sides of the fibre cable during the measurement for linear differential attenuation.

**Figure 15:**
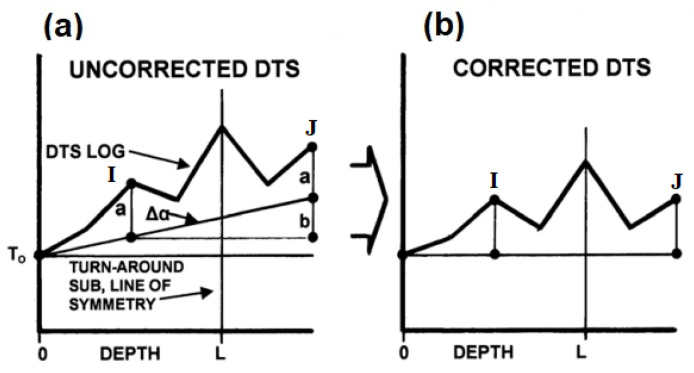
Calibration for a Linear Differential Attenuation Duplexed Single Ended System [86].

Figure 15 shows a representation of a linear differential attenuation profile for a duplexed single-ended configuration. The temperature reading for the DTS log is higher at one end of the fibre than the other hence the line of symmetry for the log does not correlate. Calibrating this system involves locating two points on the line at the same depth. Using the plane of symmetry about the turnaround sub, an equal amount of fibre is taken up the hole from the point of symmetry. As shown in the schematics with the assumption that there is no overstuff (excess fibre length over tube length) and the distances “I” to the black dot as shown in Figure 15a and “J” to the same dot are both equal from the point of symmetry [86]. The points where the attenuation line intercepts the DTS log at I and J is used as a reference. At the same depth, the upgoing fibre has greater temperature than downgoing fibre and the slope can be acquired from b/2I which is the differential attenuation correction factor. The amount “b” in Figure 15 is the difference between the fibre temperatures at the two reference points. A true DTS log can be reconstructed when the calculated slope is corrected on the DTS instrument. This computation is under the assumption of linear attenuation over the entire fibre length. This method is a step improvement from the single-ended configuration considering that an additional sensor is not needed down-hole to calibrate the system for accurate temperature measurement. However, the flaw with this configuration is that this is mainly based on the assumption that attenuation is always linear and cannot be applied in fibre cable with nonlinear differential attenuation.

#### 4.1.3. Double Ended Configuration

Double-ended fibre installation is similar to the duplex single-ended configuration other than the fact that the returning fibre cable from the turnaround sub terminates at the surface and is connected to the DTS instrument box. Here the instrument box makes observation from both ends of the fibre cable. This method offers the possibility of calibration for accurate survey in the presence of nonlinear differential attenuation along the length of the fibre cable hence unlike the single-ended, in a duplexed configuration, the entire length can be considered for correction [105]. The double-ended system can effectively cancel out losses and anomalies from data trace by interrogating the fibre from both ends and removing automatically the attenuation error along the total length of the fibre.

**Figure 16:**
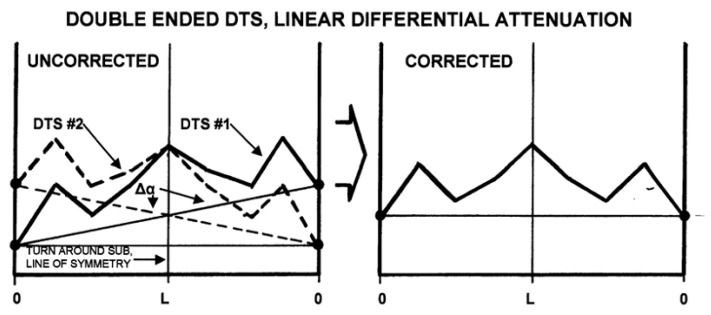
Linear differential attenuation of Double-ended Fibre Cable Configuration [86].

Figure 16 is a representation of two ended duplex configurations with a linear differential attenuation. In calibrating this system the turnaround sub line as shown in the figure below is located at the centre of the DTS survey. Represented by the black dot is the base line indicating the temperature at each end of the fibre cable connected to the DTS system at which the survey is initiated at depth zero. The average of the two curves for the differential attenuation is the correction factor for the DTS survey.

**Figure 17:**
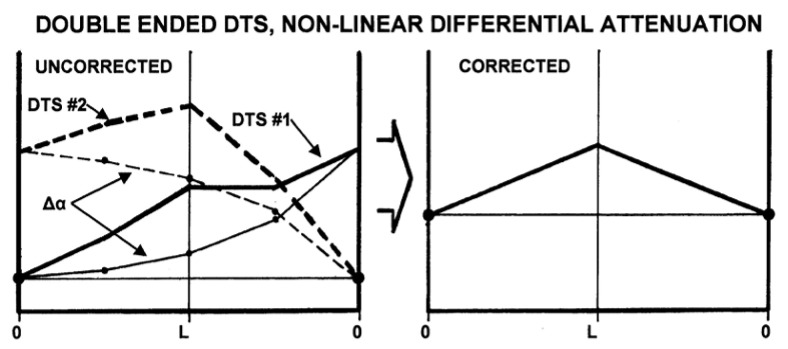
Non-linear differential attenuation of Double-ended Fibre Cable Configuration [86].

In the event of a non-linear differential attenuation condition, the double-ended system can effectively handle this issue. From Figure 17, the attenuation rate for the double-ended configuration is non-linear; the temperature is observed to have risen from one end of the fibre to the other. Hence, the average of the curve created by the DTS measurement on the fibre from the reference temperature to the temperature at the returning end of the fibre is the resultant corrected curve. The installation in the well is more complicated with added algorithm complexity and often noisier signal close to the DTS system [105].

### 4.2. Typical Oil and Gas Well Installation of Distributed Sensing System

**Figure 18:**
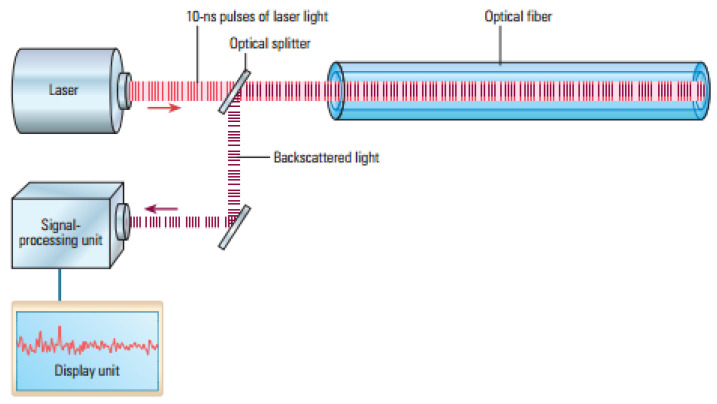
Distributed Temperature Sensing System.

To fully understand how DTS works, it is imperative to recognise the various components that make up this system. Figure 18 shows a typical distributed temperature sensing system. The DTS instrument box comprises three basic components: a laser source, fibre cable sensors and an analysis unit. The laser source and analysis unit are usually contained in one device. This laser pulse is directed to the fibre line by the directional coupler. For calibration purposes, the fibre line is passed through an oven or bath to obtain a known reference temperature. As explained earlier the light pulse moves along the fibre line, backscattering is initiated in Rayleigh, Brillouin and Raman bands of the light spectrum. The optical filter in the instrument box or DTS system screen out the Rayleigh, Brillouin and background noise from the captured signals and retains just the Raman signal for processing. After readings have been acquired for temperature measurement, a temperature vs. depth and time plot is graphically represented for analysis.

The DTS instrument box has a laser source that is capable of launching multiple laser light pulses into the fibre-optic line through the directional coupler [105].

**Figure 19:**
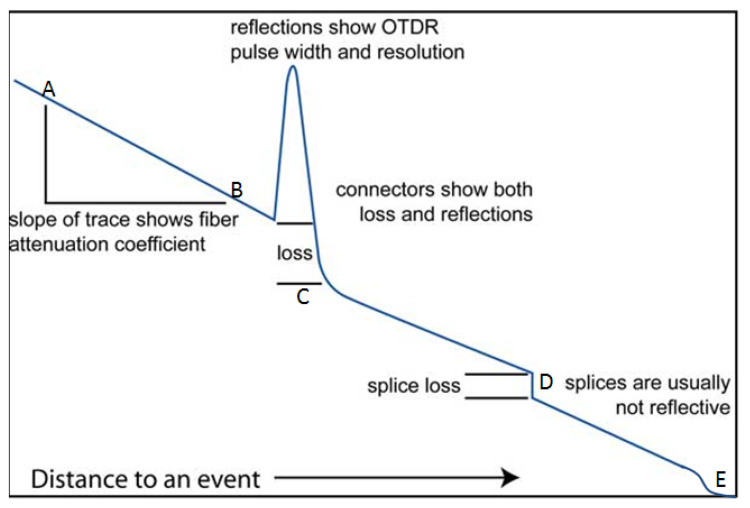
Optical Time Domain Reflectometer (OTDR) trace for Fibre integrity with distance and scattered intensity along x and y-axes, respectively.

A typical OTDR trace is shown in Figure 19 with the y axis representing the relative scattered power or intensity (dB) and *x* axis representing the distance (km); it is a graphical representation of the scattered intensity over distance along the fibre cable. As shown in the diagram, linear attenuation can be seen from point A to B indicating that the attenuation rate along the fibre at that section is constant. Point C is an indication of the reflection and loss on a connector while D is an indication of the splice. It is important to note that the attenuation rate can be affected by splice or connectors along the fibre cable. Section E is an indication of a damaged part of the fibre cable where attenuation is large and fluctuating. The method of optical fibre installation in the well is briefly discussed below.

#### 4.2.1. Retrievable Installation

**Figure 20:**
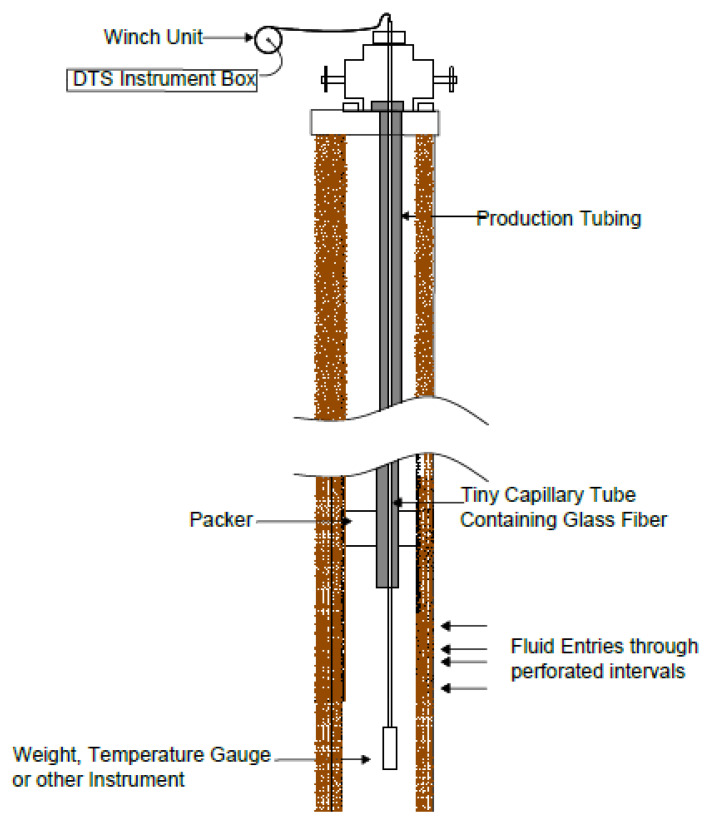
Retrievable fibre cable installation [86].

In the retrievable installation method (Figure 20), typically, the fibre cable is run into an existing well which is quite similar to a slick line or wire-line run. Housed in a protective steel capillary tube, the fibre is coiled into a steel reel drum, with a weight or sensor attached to the end of the fibre lowered into the well. After taking measurement the line can be retrieved. In a horizontal well, coil tubing can be used as a medium through which the fibre cable takes measurement in the well. The fibre cable is specially built into the coil tubing for this kind of operation [86].

#### 4.2.2. Semi-Permanent Installation

**Figure 21:**
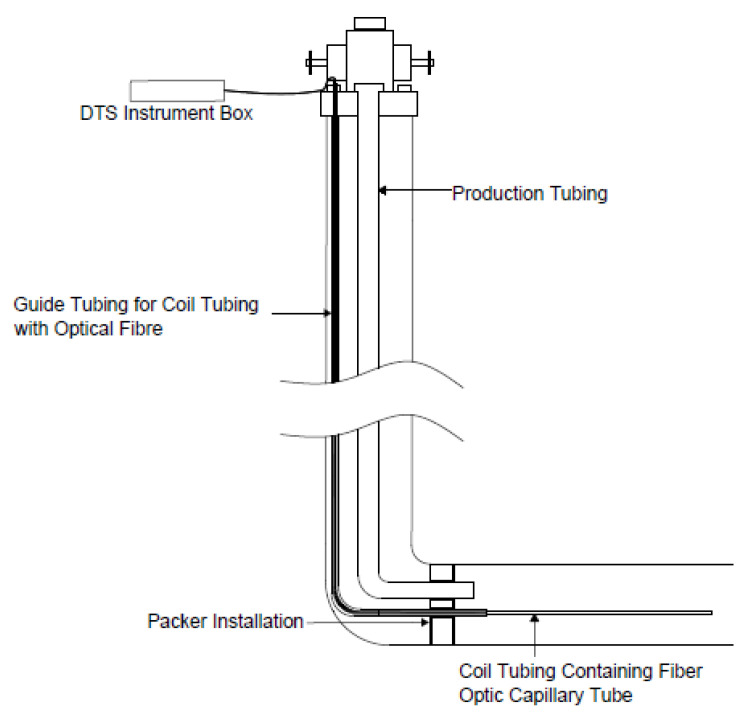
Semi Permanent Installation with coil tubing [86].

In semi-permanent installation (Figure 21), coil tubing can be permanently installed on the well head and a guide string runs in the well to Total Depth (TD) to convey the coil tubing and fibre cable. Measurement can be carried out when demanded by pumping the fibre line down through the already run in guide string via the tubing. When the measurement is carried out, the fibre and coil tubing can be pulled out of the well [86].

**Figure 22:**
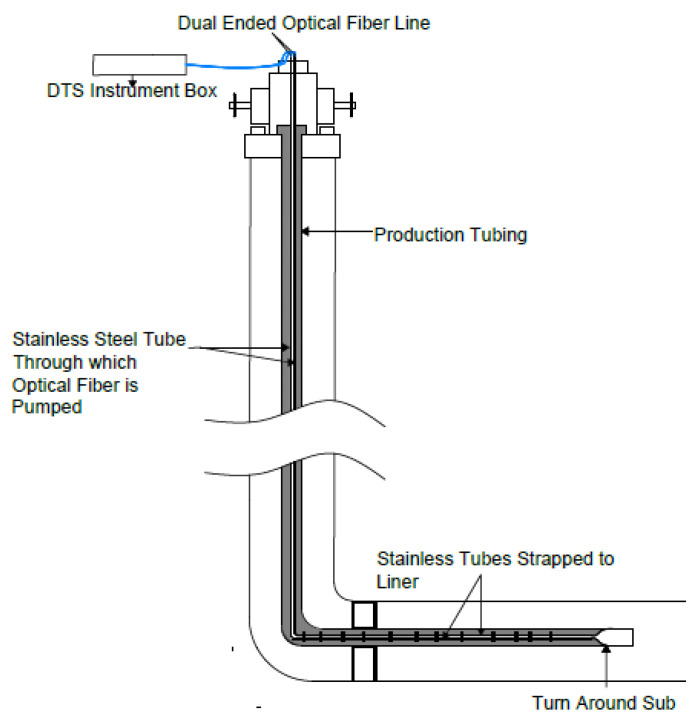
Semi-Permanent Installation with special guide tubing [86].

Another Semi-Permanent method practised in the industry involves the installation of a stainless-steel capillary tube strapped along the outer diameter of the production tubing as shown in Figure 22. The capillary tube usually with a diameter of 0.25” or smaller is installed permanently in the well [86]. The capillary tubing serves as a medium through which the fibre cable is pumped into the well for the DTS survey. This is often used for monitoring submersible pumps, gas lift mandrel operation, well flow profile, etc. Special packers are designed to allow the capillary tube to pass through the packer preventing leakage into the annulus of the production tubing.

#### 4.2.3. Permanent Installation

**Figure 23:**
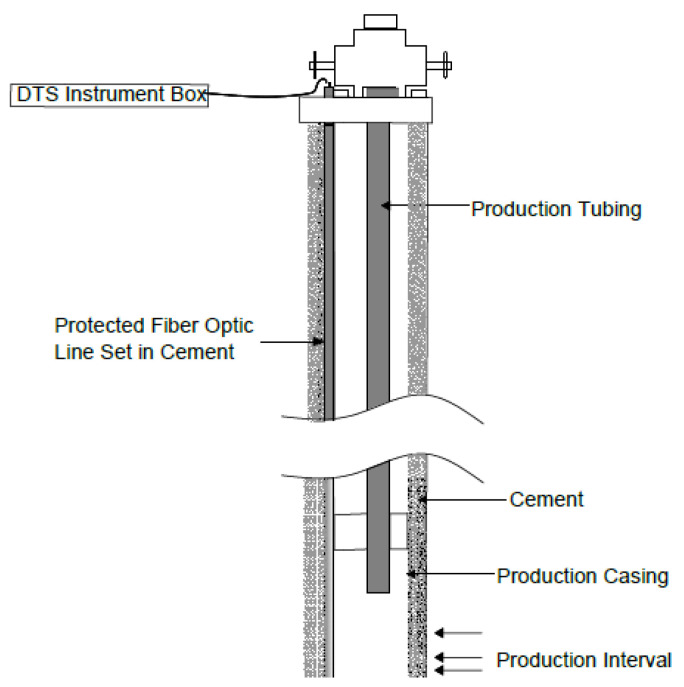
Permanent DTS Installation [86].

Fibre cable can be installed permanently in the well by running it on the surface of the casing, liner or production tubing in the well as shown in Figure 23. When the fibre is strapped to the surface of the casing, it is either cemented in place as in the case of cased-hole completion or permanently strapped on the casing surface with specialised clamps for protection in open-hole completion. Some permanent installations use a non-retrievable stainless-steel tube installed and cemented along with the casing [86].

## 5. Optical Fibre-Based Multiparameter Sensors

Different kinds of losses like the leakage or mode confinement losses experienced by traditional optical fibres is a major limitation, especially when looking for long-distance fibre-optic remote sensing applications [109,110]. Furthermore, noises are frequently generated within the fibre-optic sensing system and also from the environment which greatly influences the noise floor performance and the weak signal detection especially in largescale quasi-distributed sensing network [111]. Hence, to compensate for these noise effects within the sensing system and also the losses in the fibre sensors, advanced techniques are required for various O&G remote sensing applications. Moreover, the oil industry is keen on developing multi-point or distributed multimodal sensors, for the condition monitoring of critical parameters like pressure, temperature, vibration, strain, etc. [112,113].

Multiparameter sensors provide versatile sensing solutions, enabling miniaturization of the sensors and also bringing new sensor functionalities with improved measurement performances for sensing different critical parameters [114]. This part of the review will focus on optical fibre-based sensor configurations suitable for multi-parameter sensing and will also look into various approaches for improving the SNR of these fibre sensors for O&G application.

Among the different optical fibre-based sensors, FBG sensors have several distinct advantages and are competent to sense almost all physical parameters such as temperature, strain, pressure, vibration, etc., and also offer multi-point sensing with appreciably good range. Specific advantages of FBG sensors over other types of fibre-optic sensors are: They give wavelength encoded measurements and hence information is not susceptible to light power fluctuations [15]. Multiple gratings can be inscribed onto the same fibre, taking advantage of the wavelength multiplexing capability [115]. This enables them to read many numbers of sensors on very few fibres, resulting in reduced cabling requirements and easier installation. Multi-point or quasi-distributed sensing can be achieved in a cost-effective and compact manner [116]. The sensor responds to strain and temperature in a linear and additive manner [117]. A major limitation of the FBG sensor is its cross-sensitivity, as it responds to multiple parameters like temperature and strain in a coupled fashion [52]. Consequently, the effects of these parameters need to be separated out from each other, in order to make measurements simultaneously or separately. Hence, the existing FBG technology needs to be adapted to overcome its technical difficulties, for ensuring better performance for the oil industry.

Many techniques have been already explored for the discrimination of physical parameters in FBG sensors. A common approach is to engage two FBGs in close proximity, wherein one is the sensing FBG and the other is the reference FBG which is kept isolated from one of the parameters [118]. Furthermore, the sensor and reference gratings can be on the same or different optical fibres as well [119]. Another method is to employ two FBGs operating at different Bragg wavelengths, responding distinctly to different parameters [120]. Besides this, measurement of wavelength shift in two FBGs, showing different responses to parameters like temperature and strain enables simultaneous measurement of the two parameters [121]. An alternative technique proposed was, FBGs inscribed on different-diameter fibres, giving distinct strain responses and same temperature responses [40,122].

However, utilizing multiple FBGs for distinguishing various physical parameters restrict the number of sensors that can be deployed down-hole. The major factor deciding the number of FBG sensors that can be designed on the optical fibre is the wavelength range, which is also termed as the “wavelength window”. Individual sensors need room to vary up and down in wavelength corresponding to changes in their environmental parameters [123]. One method to accommodate more FBG sensors on the optical fibre is to narrow down the spectral width of its reflected signal. Furthermore, FBG sensors also experience low SNR values with increasing transmission length [124,125]. Hence, in order to improve SNR, the amplitude of the FBG reflectivity signal need to be increased and the noise caused by crosstalks from adjacent channels need to be minimized [126,127].

The intrinsic pressure sensitivity of FBGs is very low, therefore polymer coatings are required to improve its sensitivity [128]. These polymer coatings [convert transverse pressure on the sensor into longitudinal strain. For example, polyamide [25,26,27] and acrylate [28] coatings considerably increase the sensor sensitivities to temperature and strain.

Major challenges of multi-point multimodal FBG sensing are listed below: (i) Cross-sensitivity—it is a key problem in FBG sensors, where one physical parameter (temperature or strain) influences the value of the other. (ii) Cross-coupling—nonlinear coupling of sensor parameters makes it hard to decouple measurands of different physical variables. (iii)Transmission and sensing range—the range of the sensor is very important because physical parameters are to be measured from ultra-deep oil wells. (iv) SNR–SNR can be improved by increasing the reflected signal amplitude and by reducing the signal attenuation and crosstalks. (v) Number of sensors—the finite optical spectrum limits the number of sensors that can be designed. More sensors are required to achieve multi-point or distributed sensing down-hole. (vi) Sensitivity—enhanced sensitivity to physical parameters like pressure and temperature can be achieved with suitable polymer coatings.

Although FBGs are capable of sensing almost all physical parameters like temperature, strain, pressure, vibration, etc.; however, they respond to multiple sensing parameters in a coupled fashion. Therefore, to measure these physical parameters separately or both simultaneously, the effects of temperature and strain need to be decoupled from each other. Therefore, multi-parameter measurements become important, as it allows to reduce the size, cost and complexity of the sensing system, and also provides parameter discrimination in situations where cross-sensitivity is a critical issue [129]. Additionally, more sensors are required down-hole with a longer sensing range for effective remote monitoring of the oil wells. However, this results in higher signal attenuation, crosstalks and losses. In order to avoid this situation, there is a need to enhance the sensing signals and also reduce fibre losses. Considering the factor that the optical spectrum is limited, so the broad reflected spectrum limits the number of sensors that can be designed. Therefore, in order to include more sensors, the spectral width of the reflected signal needs to be reduced.

Even though intensive research studies have been carried out on specialised fibres like PCFs [130,131] and FBGs for many years, not much work has been reported utilizing their synergy in O&G sensing applications. PCF-FBG-based sensor is capable of differentiating the effects of different parameters like temperature and strain [132]. Their combination can improve the overall performance of the sensor system in terms of power, energy scaling and discrimination of cross-sensitivities [133,134]. The reflectivity peaks from the PCF-FBG sensor have inconsistent sensitivity to external parameters which helps to attain multi-parameter measurement simultaneously, offering good stability and a wide range of broadband tuning [135,136,137,138].

The main advantage of PCF is their high light confinement characteristics which are otherwise difficult to achieve in ordinary conventional fibres. LCPCF (Liquid crystal PCFs) open new perspectives in sensing applications [139]. With the addition of liquid crystals onto the PCF air holes, its output signal experiences a wavelength shift corresponding to the variation in physical parameters like temperature [140,141]. Liquid crystals also provide means of achieving active control over PCF propagation and polarisation characteristics [142]. The thermal and electrical tuning capabilities of LCPCFs along with their unique spectral and polarisation properties opens up their possibilities for multi-parameter fibre-optic sensing. Furthermore, through PCF nanostructuring, ultra-low confinement losses can be achieved in a large wavelength region [143,144,145,146]. The guidance properties of the PCF, with a radially periodic cladding structure of concentric high and low-index layers, can be improved by optimising its effective refractive index [147]. A core with a higher refractive index facilitates higher light confinement [148] which in turn increases the propagation distance of the light signal. One approach to manipulate the core refractive index is through rare-earth doping [149,150,151]. The refractive index of glass increases with increasing rare earth concentration [152]. Another offshoot advantage of doping the fibre core with rare earth elements is its fluorescence and Raman signatures [153]. For different laser excitations, the doped glasses generate specific Raman and fluorescent emissions corresponding to each rare earth element [154]. These Raman and fluorescent emissions can be utilized for sensing applications as they are sensitive to physical parameters like temperature and also to chemicals [155,156,157,158]. In addition, Raman and fluorescent emissions from the rare earth doped core PCF-FBG sensor facilitates multi-parameter sensing. Raman-based sensing technique also facilitates distributed temperature sensing [159], which is very beneficial while looking for the complete temperature profile of the oil well. To sum up, optical fibre sensing technologies are promising solutions for smarter sensing systems in the offshore oil industry.

The new multimodal sensor configuration is expected to be a multi-wavelength time-multiplexed fibre-optic sensing system capable of multi-point strain sensing, distributed temperature sensing and key point chemical or temperature measurements. Quasi-distributed FBGs with multi-wavelength-based detection enhances the capabilities of the sensor combination, enabling sensing of critical parameter like strain at multiple points. Microstructuring and liquid crystal infiltrations of cladding holes enhance the signal intensity from the sensor configuration and also enables wavelength tunability required for the rare-earth-based sensors. Rare earth doping of PCF core further enhances the signal intensity and generates Raman signatures useful for Raman-based DTS systems. Furthermore, localised rare-earth doping enables multi-point fluorescence sensing of critical parameters like temperature or chemicals. The multimodal sensor can be interrogated using different optical pulses with different frequencies or wavelengths. A multi-wavelength light source system can be used consisting of different laser wavelengths needed for probing the quasi-distributed FBGs, multi-point rare earth fluorescence sensors and Raman DTS.

## 6. Outlook

The various distributed sensing technologies (DTSS, DAS, DPS, DCS, etc.), FBG sensors, interferometry-based sensors are all efficient for single parameter measurement—either temperature, pressure, vibration or strain. For the condition monitoring of multiple critical parameters like high pressure, high temperature, vibration, etc., the oil and gas industry is intently looking for multi-point or distributed multimodal sensors. Multimodal sensors will provide a flexible sensing solution, enable miniaturization of the sensors, and add new functionalities with improved measurement capabilities for sensing various important parameters. Furthermore, multimodal fibre-optic sensor configuration will be capable of sensing multiple parameters from different locations. An integrated fibre-optic sensor configuration, combining features of LCPCF, FBG and rare earth doping; extracting their molecular, atomic and vibro-rotational characteristics would be a good option for sensing multiple modalities in the oil industry.

The main limitation of existing fibre-optic sensors is they use a single wavelength region (mostly communication window around 1550 nm) for probing the parameters. The highlight of the proposed multimodal sensor is that they operate in multiple wavelength regimes, adding scope for new functionalities and sensing capabilities. Microstructured fibre is also expected to improve the light confinement characteristics which in turn enhances the signal intensity from the multimodal sensor configuration. Infusing liquid crystals into the PCF sensor will aid in the tuning of spectral bands from the communication window to other wavelength regimes (NIR and visible).

Electrochemical change induced in the PCF by infusion of liquid crystals will result in a change in the effective refractive index of cladding and also tunes the optical bandgaps. Application of electrical voltage for aligning the LC directors and subsequent refractive index changes both plays a key role in the shift of different spectral bands. Bandgap tuning enables the LCPCF sensor to operate within the photonic bands that have peak sensitivities. Liquid crystals can be infiltrated into the PCF holes via capillary effects, a technique known as vacuum-assisted LC infiltration is commonly used.

Doping the core of microstructured fibres with rare earth elements can further enhance the light confinement characteristics and also invoke fluorescence and Raman signatures when probed with suitable laser excitations. These specialised features of LCPCF can be effectively utilized in the new sensor configuration for tuning the light confinement wavelengths to suit the fluorescence and Raman spectral regions of the rare earth doped sensors.

Furthermore, integrating FBGs into the sensor configuration will enable multi-point multimodal sensing capabilities. The integrated sensor combination is expected to overcome the limitations of existing fibre-optic sensors with regards to cross-sensitivity, SNR, sensing range and multimodal sensing capability. The new sensor configuration operating in multiple wavelength regimes in a multiplexed fashion will have the potential to carry out multi-parameter sensing.

## 7. Conclusions

A detailed review of different fibre-optic sensing techniques was carried out, to identify a feasible sensing solution for the oil industry. The different classifications of the fibre-optic sensors and different types of fibre-optic sensors used for oil and gas applications were discussed. The challenges posed by the oil and gas harsh environments were discussed. The technical challenges associated with the different fibre-optic sensing technologies, especially the most commonly used FBG sensors and distributed fibre-optic sensors were described in detail. Moreover, a thorough review leading to a new idea for multimodal sensing using a novel fibre-optic configuration for oil and gas applications was discussed. Lastly, to improve the performance of the multimodal sensor in terms of its SNR, different approaches and techniques were identified. The proposed configuration of the novel multimodal fibre-optic sensor consisting of rare-earth-doped microstructured–FBG sensor is expected to overcome the technical difficulties and challenges of the existing sensors in the O&G industry. The integrated sensor combination is likely to overcome the limitations of FBG sensors with regards to cross-sensitivity, SNR, sensing range and multimodal sensing capability. The new sensor configuration operating in multiple wavelength regimes in a multiplexed fashion is competent to perform multi-parameter sensing.

## Data Availability

Not applicable.
